# Management of Infective Endocarditis Complicated by Ruptured Cerebral Mycotic Aneurysm: A Case Report

**DOI:** 10.1002/ccr3.73429

**Published:** 2026-08-28

**Authors:** Shogo Oyama, Junpei Yamauchi, Satoshi Arimura, Kosuke Saku, Tomomitsu Takagi, Satoshi Hoshino, Takayuki Abe, Yoko Matsumura, Michio Yoshitake, Ryuichi Nagahori, Ko Bando, Takashi Kunihara, Gota Nagayama, Yuichi Murayama

**Affiliations:** ^1^ Department of Cardiac Surgery The Jikei University Hospital Tokyo Japan; ^2^ Department of Neurosurgery The Jikei University Hospital Tokyo Japan

**Keywords:** cerebral mycotic aneurysm, infective endocarditis, mitral valve replacement, multidisciplinary team, subarachnoid hemorrhage

## Abstract

This case highlights multidisciplinary decision‐making and the timing of staged surgical intervention in infective endocarditis complicated by intracranial hemorrhage. Early cardiac surgery following neurosurgical treatment may be considered in carefully selected patients.

## 1 Introduction

A 73‐year‐old Japanese man with infective endocarditis developed a ruptured cerebral mycotic aneurysm with subarachnoid hemorrhage. Urgent neurosurgical intervention was followed by mitral valve replacement after confirming neurological stability. This case highlights multidisciplinary decision‐making and the timing of staged surgical intervention in complex infective endocarditis.

## 2 Case History/Examination

A 73‐year‐old man had previously undergone mitral valvuloplasty, tricuspid annuloplasty, and the Maze procedure in 2012 for mitral regurgitation, tricuspid regurgitation, and atrial fibrillation. In February 2021, he presented with palpitations, fatigue, and dyspnea. On admission, he had severe bradycardia (40 beats/min), acute decompensated heart failure due to mitral regurgitation, and acute renal failure (creatinine 5.42 mg/dL; potassium 7.5 mmol/L).

**FIGURE 1:**
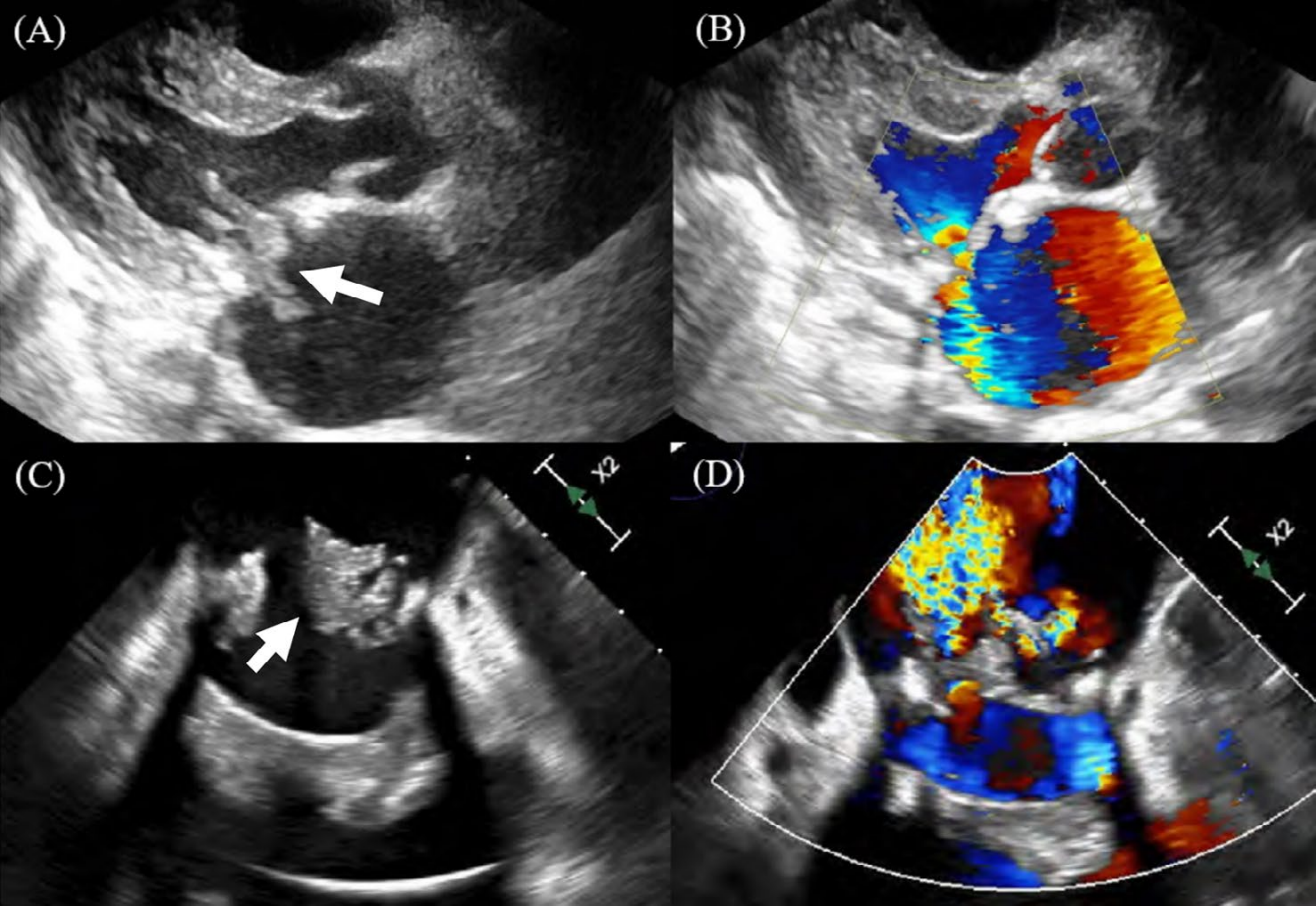
Transthoracic and transesophageal echocardiographic findings. (A, B) Transthoracic echocardiography demonstrating a mobile vegetation attached to the anterior leaflet of the mitral valve (arrow) with severe mitral regurgitation. (C, D) Transesophageal echocardiography showing generalized thickening of the mitral valve and a vegetation measuring > 10 mm in diameter (arrow).

A temporary pacemaker, Swan–Ganz catheter, and hemodialysis catheter were inserted immediately, and inotropic support with continuous renal replacement therapy was initiated. Transthoracic and transesophageal echocardiography revealed severe mitral regurgitation with a large, highly mobile vegetation exceeding 10 mm in diameter (Figure 1). The patient also exhibited mild motor aphasia and higher‐order brain dysfunction, with a National Institutes of Health Stroke Scale score of 1. Blood cultures obtained on admission grew *Abiotrophia defectiva* . Empirical antimicrobial therapy with intravenous ceftriaxone and vancomycin was initiated on admission. After susceptibility results became available, therapy was streamlined to ceftriaxone 2 g every 12 h. Because of fever and skin rash during treatment, ceftriaxone was replaced with ampicillin, which was continued to complete a total of six weeks of intravenous antimicrobial therapy.

**FIGURE 2:**
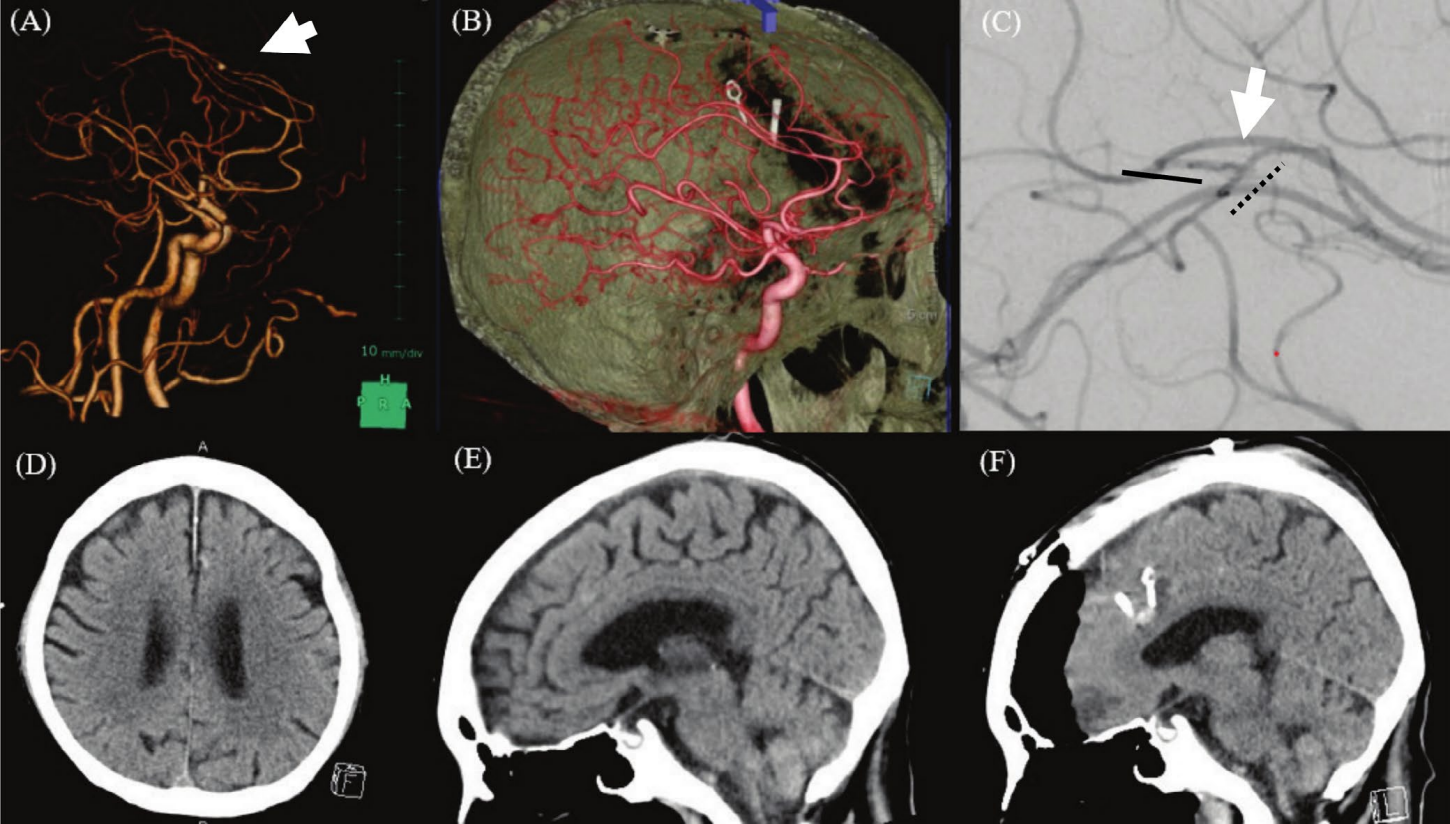
Neuroimaging findings of the cerebral mycotic aneurysm. (A) Contrast‐enhanced computed tomography angiography before craniotomy demonstrating a saccular aneurysm measuring approximately 4 mm in the left anterior cerebral artery (arrow). (B, C) Cerebral angiography after trapping and bypass surgery demonstrating a patent end‐to‐side anastomosis between the right pericallosal artery and the left callosomarginal artery. In panel C, the right pericallosal artery (solid line), left callosomarginal artery (dashed line), and anastomotic site (arrow) are indicated. (D, E) Preoperative non‐contrast computed tomography demonstrating a high‐density area suggestive of subarachnoid hemorrhage adjacent to the aneurysm. (F) Postoperative computed tomography after trapping and bypass surgery showing no progression of intracranial hemorrhage.

Contrast‐enhanced computed tomography angiography demonstrated a saccular aneurysm in the distal anterior cerebral artery, and digital subtraction angiography confirmed a fragile aneurysmal lesion consistent with a cerebral mycotic aneurysm (Figure 2). Non‐contrast computed tomography showed subarachnoid hemorrhage. Additional findings included renal and splenic infarctions. After multidisciplinary discussion involving cardiologists, cardiac surgeons, neurosurgeons, neurologists, and infectious disease specialists, urgent neurosurgical intervention was prioritized.

## 3 Methods (Differential Diagnosis, Investigations, and Treatment)

Given the ruptured status of the aneurysm and the high risk of rebleeding, urgent neurosurgical intervention was performed. Preoperative neuroimaging demonstrated morphological features suggestive of an infectious aneurysm in the distal anterior cerebral artery (Figure 2). Open neurosurgical treatment was selected as one reasonable option to achieve definitive hemorrhage control in this specific clinical context. The aneurysm was treated by trapping combined with bypass surgery. An end‐to‐side anastomosis was created between the right pericallosal artery and the left callosomarginal artery to maintain distal cerebral perfusion. Postoperative computed tomography confirmed the absence of intracranial rebleeding.

**FIGURE 3:**
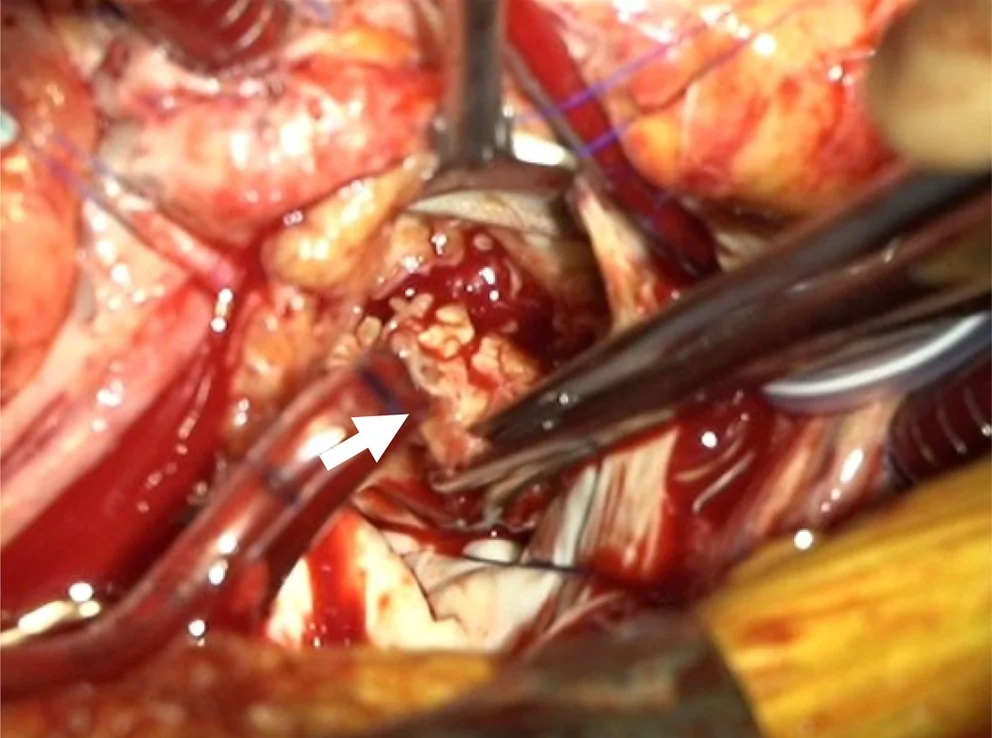
Intraoperative findings during cardiac surgery. Intraoperative view demonstrating severe destruction of both mitral valve leaflets due to active infective endocarditis. A mobile vegetation attached to the anterior leaflet is shown being grasped with surgical forceps.

Following confirmation of stable postoperative neurological findings and the absence of intracranial hemorrhage on follow‐up imaging, cardiac surgery was performed on the day after neurosurgical intervention. Re‐sternotomy and a transverse transseptal approach revealed extensive destruction of both mitral valve leaflets due to active infective endocarditis, precluding repeat valvuloplasty (Figure 3). Mitral valve replacement with a bioprosthetic valve was therefore performed.

## 4 Conclusions and Results (Outcome and Follow‐Up)

The postoperative course was uneventful. The patient was extubated on the following day without neurological deterioration. On postoperative day 56, the patient was transferred to another hospital for rehabilitation. No apparent motor deficits were observed; however, mild higher cortical dysfunction persisted. No recurrence of infection was observed during follow‐up.

## 5 Discussion

Cerebral mycotic aneurysm is a rare but potentially fatal complication of infective endocarditis, particularly when rupture results in subarachnoid hemorrhage [1, 2, 3]. Although endovascular techniques have expanded treatment options, limitations in achieving durable infection control have been reported in cases with extensive arterial wall involvement or fragile aneurysmal morphology [2, 4].

In the present case, preoperative neuroimaging demonstrated a ruptured distal anterior cerebral artery aneurysm with features consistent with an infectious lesion (Figure 2). Given the high risk of rebleeding, urgent neurosurgical intervention was prioritized. This approach may not be generalizable to all patients with infective endocarditis complicated by intracranial hemorrhage; rather, it represents one possible management strategy in a carefully selected patient.

The optimal timing of cardiac surgery after intracranial hemorrhage remains controversial. While delayed surgery has traditionally been recommended to reduce the risk of neurological deterioration, current guidelines emphasize individualized assessment based on neurological stability and cardiac urgency [5, 6, 7, 8]. In this patient, postoperative neuroimaging confirmed stable intracranial findings, whereas intraoperative cardiac findings demonstrated severe and irreversible destruction of the mitral valve (Figure 3), and progressive heart failure posed a substantial risk if surgery were delayed. After multidisciplinary discussion, early cardiac surgery was considered the most appropriate option.

Management decisions in this case were made through close collaboration among cardiologists, cardiac surgeons, neurosurgeons, neurologists, and infectious disease specialists, consistent with the concept of an endocarditis team [8]. This multidisciplinary framework facilitated balanced risk assessment and consensus‐based decision‐making in a complex clinical scenario. This case provides practical insights into multidisciplinary decision‐making and timing of intervention in infective endocarditis complicated by intracranial hemorrhage.

## 6 Conclusions

Management of infective endocarditis complicated by ruptured cerebral mycotic aneurysm requires individualized decision‐making. This case demonstrates that early cardiac surgery following neurosurgical treatment may be feasible in carefully selected patients after thorough multidisciplinary assessment, without implying generalizability to all similar cases.

## Author Contributions

**Shogo Oyama:** writing – review and editing, writing – original draft, investigation, resources. **Kosuke Saku:** investigation, resources. **Junpei Yamauchi:** resources, investigation. **Satoshi Hoshino:** resources, investigation. **Takashi Kunihara:** supervision. **Yoko Matsumura:** investigation, resources. **Yuichi Murayama:** supervision. **Tomomitsu Takagi:** investigation, resources. **Satoshi Arimura:** investigation, resources. **Ryuichi Nagahori:** investigation, resources. **Takayuki Abe:** investigation, resources. **Michio Yoshitake:** investigation, resources. **Ko Bando:** investigation, resources. **Gota Nagayama:** writing – review and editing.

## Funding

The authors have nothing to report.

## Ethics Statement

The authors have nothing to report.

## Consent

Written informed consent was obtained from the patient for publication of this case report and any accompanying images. A copy of the written consent is available for review by the Editor‐in‐Chief of this journal.

## Conflicts of Interest

The authors declare no conflicts of interest.

## Data Availability

All data generated or analyzed during this study are included in this published article.
